# A novel classifier combining G protein-coupled receptors and the tumor microenvironment is associated with survival status in glioblastoma

**DOI:** 10.3389/fphar.2023.1093263

**Published:** 2023-07-25

**Authors:** Chunyu Guo, Cong Yu, Weizhen Gao, Dabin Ren, Yisong Zhang, Ping Zheng

**Affiliations:** ^1^ Department of Neurosurgery, Shanghai Pudong New area People’s Hospital, Shanghai, China; ^2^ Department of Neurosurgery, Renji Hospital, Shanghai Jiaotong University, Shanghai, China

**Keywords:** G protein-coupled receptors (GPCRs), tumor microenvironment (TME), glioblastoma (GBM), T cells, classifier

## Abstract

**Background:** Numerous studies have highlighted the crucial role of G protein-coupled receptors (GPCRs) in tumor microenvironment (TME) remodeling and their correlation with tumor progression. However, the association between GPCRs and the TME in glioblastoma (GBM) remains largely unexplored.

**Methods:** In this study, we investigated the expression profile of GPCRs in GBM using integrated data from single-cell RNA sequencing and bulk sequencing. Surgical samples obtained from meningioma and GBM patients underwent single-cell RNA sequencing to examine GPCR levels and cell-cell interactions. Tumor microenvironment (TME) score is calculated by the infiltrated immune cells with CIBERSORT.

**Results:** Our findings revealed a predominantly increased expression of GPCRs in GBM, and demonstrated that the classification of GPCRs and TME is an independent risk factor in GBM. Patients with high GPCR expression in the tumor tissue and low TME score exhibited the worst outcomes, suggesting a potentially aggressive tumor phenotype. On the other hand, patients with low GPCR expression in the tumor tissue and high TME score showed significantly better outcomes, indicating a potentially more favorable tumor microenvironment. Furthermore, the study found that T cells with high GPCR levels displayed extensive cell-cell connections with other tumor and immune cells in the single cell RNA analysis, indicating their potential involvement in immune escape.

**Conclusion:** In conclusion, GPCRs in combination with TME classification can serve as prognostic markers for GBM. GPCRs play an essential role in tumor progression and the TME in GBM.

## 1 Introduction

Glioma, including glioblastoma (GBM), is a highly malignant cancer of the central nervous system with a low survival rate (Wijethilake et al., 2021). Standard therapies have limited effectiveness (Olgun et al., 2021), resulting in a high risk of recurrence and metastasis (Mohme et al., 2020). Therefore, there is an urgent need to identify novel therapeutic targets for GBM treatment.

Orphan G protein-coupled receptors (GPCRs) are a vast superfamily of cell surface receptors, which have been implicated in tumor growth and metastasis (Feigin, 2018). However, their role in glioma has not been adequately assessed (Trzaskowski et al., 2012). Upon ligand binding, GPCRs undergo conformational changes (Gookin et al., 2008) and activate downstream signaling pathways. Abnormal activation of GPCRs can lead to aberrant cell proliferation and metastasis in cancer (Arang and Gutkind, 2020).

The tumor microenvironment (TME) has emerged as a crucial player in cancer progression, particularly involving immune cells (Takasugi et al., 2022; Tamai et al., 2022). Tumor-infiltrating lymphocytes (TILs), including T cells, are important cytotoxic fractions within the TME (Sugimura and Chao, 2022). Understanding the function and mechanisms of T cells in the TME can provide valuable insights for glioma treatment, especially immunotherapy (Boissonnas and Combadière, 2022; Li et al., 2022). Despite numerous clinical trials targeting T cells (Andersen et al., 2022; Wang et al., 2022), such as chimeric antigen receptor T-cell immunotherapy (CAR-T) therapy (Berdeja et al., 2021; Fowler et al., 2022; Hu et al., 2022; Ottaviano et al., 2022; Qi et al., 2022; Sallman et al., 2023), the outcomes in GBM immune therapy have been unsatisfactory (Cloughesy et al., 2019; Song et al., 2020; Hajji et al., 2022). The limited success of immunotherapy in GBM treatment may be attributed to the internal heterogeneity of immune cells, including T cells (Jacob et al., 2020; Wang et al., 2020; Medikonda et al., 2021).

Previous studies have examined specific G protein-coupled receptors (GPCRs) in glioma. Notably, the expression of C-C motif chemokine receptor 5 (CCR5) has been found to increase in glioma cells and mesenchymal stem cells, correlating with poor prognosis in patients (Kouno et al., 2004; Wang et al., 2016; Laudati et al., 2017; Kranjc et al., 2019; Novak et al., 2020). Additionally, glioblastoma stem cells have been found to express the receptor C-X-C receptor type 4 (CXCR4), which interacts with the chemoattractant stromal-derived factor-1α (SDF-1α). Inhibition of both CXCR4 and SDF-1α can disrupt the niche of cancer stem cells in glioblastoma (Hira et al., 2017, 2020). However, the role of GPCRs in TME of glioma remains largely unexplored.

This study aims to investigate the function and mechanisms of T cells in the tumor immune microenvironment (TIME) of GBM, particularly focusing on the role of GPCRs. The researchers use single-cell RNA sequencing to elucidate the expression profile and interactions of GPCRs and immune components in GBM TME. This research can provide valuable insights into the potential involvement of GPCRs in GBM progression and immune responses, ultimately aiding in the development of novel therapeutic approaches for GBM treatment, including immunotherapy.

## 2 Methods

### 2.1 Data download

The TCGA-GBM dataset was obtained from the UCSC Xena website (https://xenabrowser.net/datapages/). The Chinese Glioma Genomic Atlas (CGGA) dataset was acquired from the CGGA website (http://www.cgga.org.cn/).

### 2.2 Data preprocessing and GPCR genes screening

The TCGA-GBM dataset which have 169 GBM patients and five healthy controls underwent preprocessing using the Affy package in R for normalization and RMA correction. To address the batch effect between the TCGA-GBM dataset and CGGA datasets, the Limma and sva packages were utilized. From the Molecular Signatures Database (MSigDB) (https://www.gsea-msigdb.org/gsea/msigdb/cards/GOMF_G_PROTEIN_COUPLED_RECEPTOR_ACTIVITY), a total of 870 GPCR-related genes were obtained, and 18 genes associated with GPCR exhibited correlation or survival status. Differentially expressed genes (DEGs) were identified based on log2 fold change (log2FC) > 1 and adjusted *p*-value <0.05. Ethical approval for the study was obtained from the local ethical committee at Shanghai Pudong New area People’s Hospital.

### 2.3 Construction of the risk model associated with GPCR

GBM patients with effective survival data ranging from 2 days to 3,881 days were included in the study. Univariate and multiple Cox models were employed to evaluate the relationship between GPCR genes and the survival status of GBM patients. The risk score was calculated using the formula: Risk score = Coefficient of mRNA × risk genes. The Least Absolute Shrinkage and Selection Operator (LASSO) method was applied to determine the precise coefficient of each gene based on survival status. The R packages “survival” and “survminer” were utilized to compare the survival status between high- and low-risk groups. The association between survival status, GPCR genes, and the TME was assessed using KM plots, which were further validated with in-house single-cell RNA sequencing data. The TME score, calculated based on infiltrated immune cells using CIBERSORT, determined high TME (indicating a hot tumor with increased infiltrated and functional immune cells) and low TME (indicating a cold tumor with reduced immune cell infiltration). The high GPCR and low GPCR groups were classified based on the LASSO risk score. The exact TME and GPCR scores for each sample can be found in Supplementary Table S2, S3.

### 2.4 Gene set enrichment analysis

Both Gene Set Enrichment Analysis (GSEA) and the fast gene set enrichment analysis (fGSEA) were employed to quantify the score of specific gene sets in each sample. fGSEA was used to calculate gene enrichment in GBM patients with different GPCR and TME status.

### 2.5 External validation of the models

The CGGA dataset served as an external validation cohort for the risk model. GPCR scores for each patient were calculated using the formula from the TCGA model, and patients were subsequently categorized into high- and low-GPCR groups.

### 2.6 Weighted Co-Expression network analysis (WGCNA)

WGCNA analysis is a computational method for identifying co-expressed genes among different groups and identify marker genes based on the non-orientation analysis between gene sets and phenotypes. Here, WGCNA was utilized to identify gene modules related to different clusters in glioblastoma.

### 2.7 Single cell sequencing data obtained and processing

The single-cell data were obtained from our surgical samples: one is glioblastoma and the other is meningioma (serving as controls). The meningioma is a benign brain tumor without aggressive cell proliferation and invasiveness. The ethics was approved form the local ethical committee (2022-K18) and the patients’ approval has been obtained before the surgery for the willing on the surgery and taking part in this research. The consent form was also obtained from the patients and their relatives.

The exact procedure for scRNA-seq for brain tissues could be obtained from the 10X website (https://www.10xgenomics.com/cn/resources/document-library/4a0968). Fresh brain surgical samples from two brain tumor patients were collected and single-cell suspensions were isolated with the assay within 12 h after the surgery. Single-cell 3′libraries were constructed following the 10X Chromium protocol, and each single-cell 3’ library was sequenced using the Illumina Novaseq 6,000. The sequencing depth ranged from 35,000 to 50,000 mean reads per cell. And the estimated number of cells are 10,000–13,000. The median genes per cell is 2000–2,500. The database construction, sequencing, and data analysis were completed by Shanghai Ouyi Biomedical Technology Co., Ltd. Produced during high-throughput sequencing. The raw data (in fastq format), was processed using the CellRanger software from 10x Genomics. According to the data quality statistics and comparison with the reference genome, the software can distinguish cells by Recognition sequence. The high-throughput single cell Transcriptome was quantified by sequence markers and UMI markers of different mRNA molecules in each cell to obtain quality control statistical information such as high-quality cell count, gene median, and sequencing saturation. We processed the unique molecular identifier (UMI) count matrix using the R package Seurat (version 4.1.1). We first normalized the data with sctransform (SCT) in order to account for variance in sequencing depth across data points, detecting high-variance features, and stores the data in the SCT assay. Next, we carried out data quality control. We captured cells with less than 10% mitochondrial genes, and a total number of gene count ranging from 200 to 10,000, expressed in at least 3 cells were selected. Highly variable genes was set at 2000 (Zhang et al., 2021). The two samples (glioblastoma and meningioma) were integrated through single-cell transform (SCT) correction, and the uniform Manifold Approximation and Projection (uMAP) method was employed for data dimension reduction. Cellchat R package was applied to explore the cell-cell interaction between the cell clusters. The strength of cell-cell interaction is based on the total weight of ligand-receptor between different cell clusters, which includes the interaction number and communication probability (Jin et al., 2021). The monocle method was used for pseudo-time analysis to identify the stage and gene changes of T cells. GPCR extent was compared between glioblastoma and meningioma using the addmodulescore method.

### 2.8 Pseudo-time analysis

Pseudo-time analysis was performed using the R package monocle v2.16.0 to obtain genes required for calculating pseudo-time. Differential gene expression analysis was conducted among T cells from different pathological stages. After calculating pseudo-time, differential gene expression analysis was repeated to determine genes that changed as a function of pseudo-time. The cell state containing the greatest number of S0-stage cells was considered the root state. The threshold of the q-value for multiple testing involved in the selection of differentially expressed genes (DEGs) was set at 0.01.

### 2.9 Tumor mutation burden (TMB) and genes mutations between GPCR low and GPCR high group

The maftools package (Mayakonda et al., 2018) was utilized for visualizing somatic mutations and determining the TMB of each patient. TMB status was compared between patients with high and low GPCR scores. Survival status, the expression levels of immune checkpoint molecules, and human leukocyte antigen (HLA) markers were also compared.

### 2.10 Statistical analysis

All data analyses were performed in R v4.0.3, and a *p*-value <0.05 was considered statistically significant.

## 3 Results

The schematic flow of the study was shown in Supplementary Figure S1.

### 3.1 The prognosis-related GPCR genes in GBM

**FIGURE 1:**
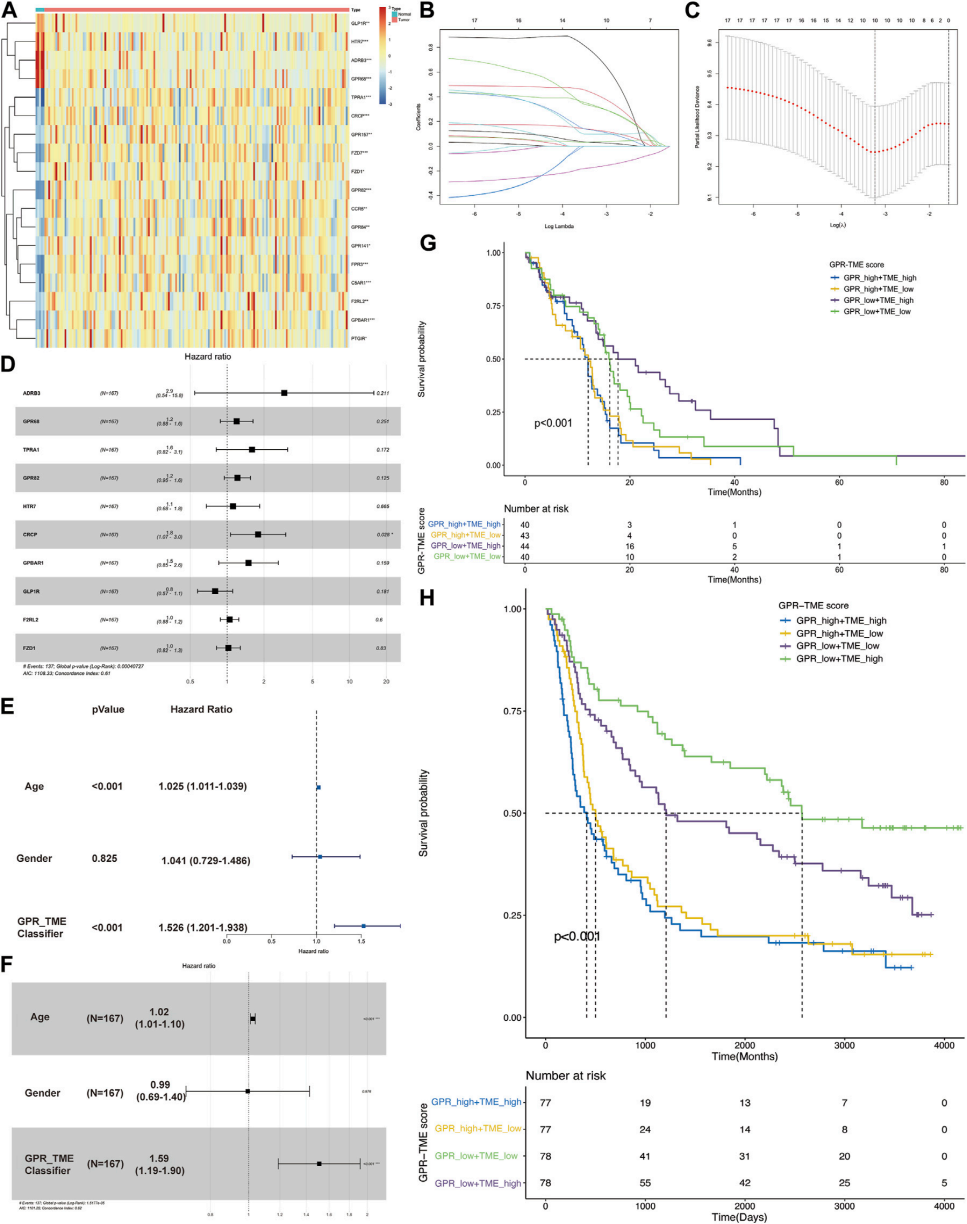
The screening of G protein-coupled receptors (GPCRs) in glioblastoma (GBM) and their association with survival status **(A)** The heatmap shows the expression level of GPCRs between the GBM and control groups **(B, C)** Lasso regression model identifies the top ten GPCRs associated with the survival status in GBM **(D)** The multiple cox analysis determines the risk factors of GPCRs in GBM **(E, F)** The univariate and multivariate Cox analysis of GPCR and TME classifier in the survival status of GBM **(G)** The KM plot depicts the survival probability among different groups from TCGA dataset **(H)** The KM plot shows the survival probability among different groups from CGGA dataset.

Figure 1A shows a heatmap demonstrating the distribution of top GPCR genes between two groups. Lasso regression analysis was conducted and identified ten GPCR genes related to survival status (Figure 1B). Using the bootstrap method, a GPCR score was constructed with the following coefficients: 1.076*ADRB3+0.180*GPCR68 + 0.468*TPRA1+0.193*GPCR82 + 0.111*HTR7+0.583*CRCP+0.402*GPBAR1+(-0.226)*GLP1R+0.046*F2RL2+0.024*FZD1 (Figure 1C). Multiple cox analysis indicated that only CRCP was an independent risk factor in GBM (Figure 1D). However, we found that the GPCR_TME classifier was also an independent risk factor with a high odds ratio of 1.51, as illustrated in Figures 1E,F. Therefore, the classifier based on high GPCR/low GPCR or high TME/low TME can be used to predict the survival status in GBM, with the worst outcome observed in patients with high GPCR/low TME and a better survival status in those with low GPCR/high TME (*p* < 0.001). This classifier was also validated in the CGGA dataset, demonstrating that in patients with high GPCR and shorter survival status, the TME did not differentiate the outcome. However, in patients with low GPCR status, the TME could further classify patients based on the survival status (Figures 1G,H).

### 3.2 Construction of Co-expression network

**FIGURE 2:**
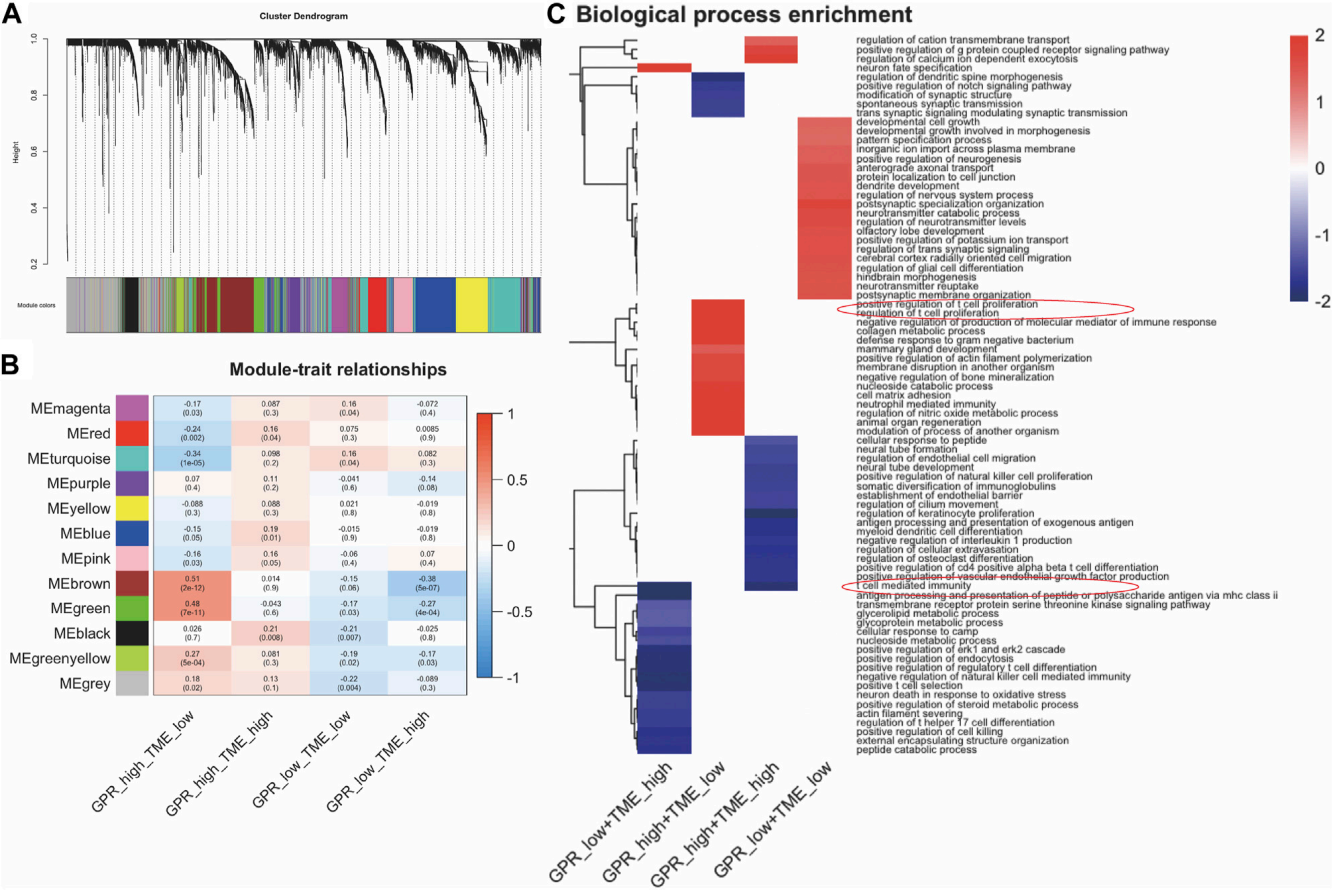
The enrichment pathway analysis among the four clusters in GBM. **(A)**. WGCNA analysis identifies modules related to GBM survival **(B)** The correlation between the modules in four groups **(C)** fGSEA analysis shows different pathway enrichments among the groups.

The co-expression network was identified using WGCNA, resulting in the identification of twelve modules (Figure 2A). The correlation between these twelve modules and the two groups was assessed by measuring the correlation between ME values and clinical features (Figure 2B). The results indicate that the brown and green modules positively correlate with the GPCR_high_TME_low phenotype (r = 0.51, p = 2e-12; r = 0.48, p = 7e-11) and negatively correlated with the GPCR_low_TME_high phenotype (r = -0.38, p = 5e-07; r = -0.27, p = 4e-04, Figure 2B). Genes in the green and brown modules were listed in Supplementary Table S4. Additionally, fGSEA indicate that the GPCR_high_TME_low group exhibit a positive correlation with positive regulation of T cell proliferation, while the GPCR_low_TME_high group show a negative association with T cell mediated immunity (Figure 2C). In the GPCR high and TME high group, there is also an association with the GPCR signaling pathway and a negative correlation with T cell-mediated immunity. Therefore, both GPCR and TME status might be involved in the T cell activity.

**FIGURE 3:**
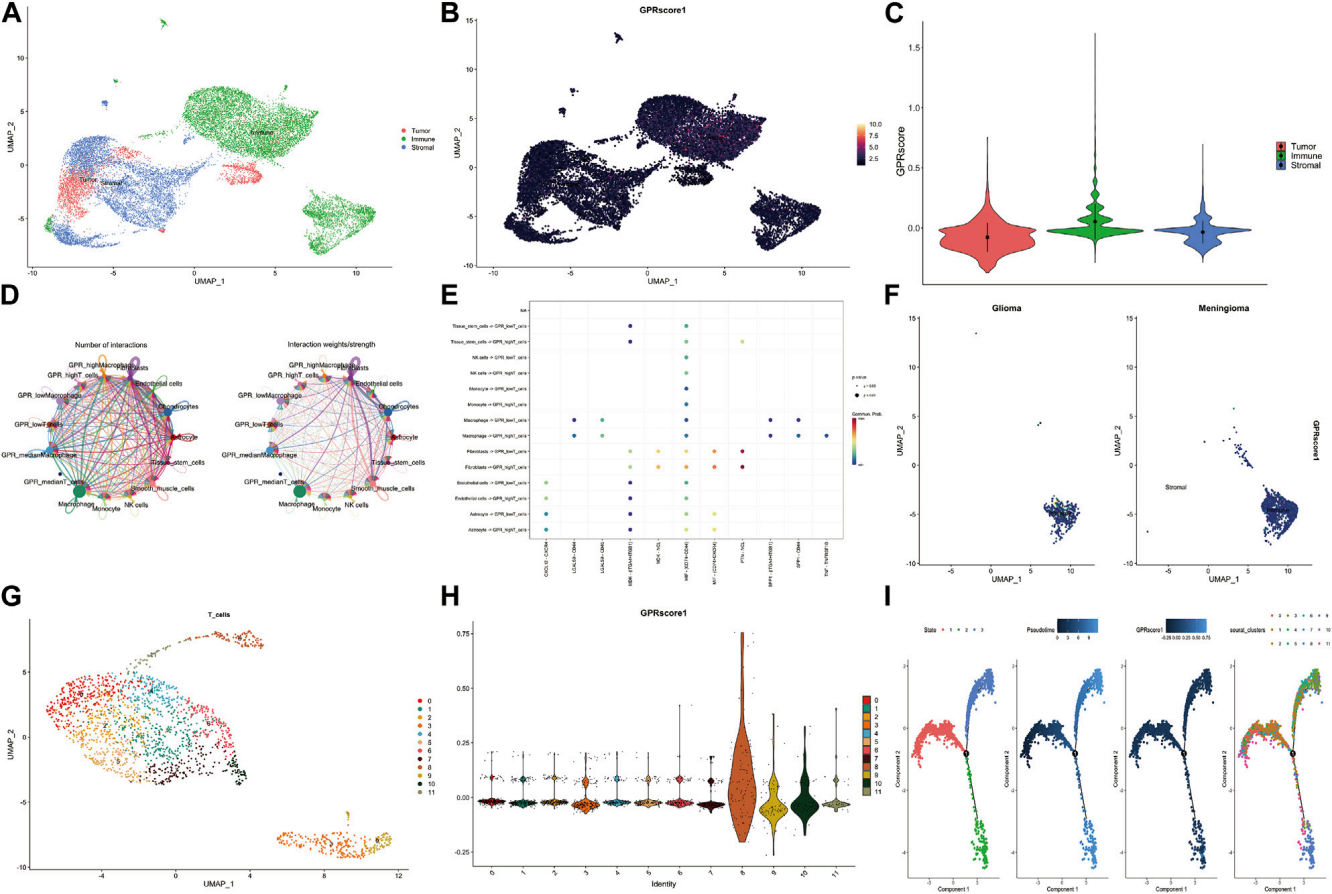
The GPCR scores between GBM and meningioma in our in-house single-cell RNA sequencing data **(A)** The specific cell clusters are shown with their markers **(B)** The GPCR scores among the tumor, immune and stromal parts assessed by addmodulescore **(C)** The comparison of GPCR score among the three groups with wilcox test **(D)** The number of interactions and interaction weights between several cell types **(E)** The bubble plot shows the relationship between cell types and ligand-receptor **(F)** The comparison of GPCR score between glioma and meningioma **(G)** The sub-cluster of T cells in the GBM **(H)** The GPCR score in different T cell sub-clusters **(I)** The pseudo-time analysis of the T cell sub-cluster.

The TCGA samples were classified into three clusters: tumor, immune, and stromal parts (Figure 3A). Further analysis using the AddModuleScore method revealed significant enrichment of GPCR genes in immune cells (Figure 3B). This finding was consistent with the violin graph presented in Figure 3C. As GPCR-related genes were primarily expressed in immune cells, we investigated cell-cell interactions at a single-cell level using the CellChat R package. The analysis revealed strong interactions between several cell types (Figure 3D). The bubble plot of the network indicated that T cells with high GPCR levels were connected to tissue stem cells via PTN/NCL, while this connection was absent in T cells with low GPCR levels. Additionally, T cells with high GPCR levels were connected to macrophages via Tnf/Tnfrsf1b, which was not observed in T cells with low GPCR levels. These results suggest that immune cells with high GPCR levels may be involved in immune escape (Figure 3E). Comparison of GPCR levels between the GBM group and the meningioma group, based on in-house sc-RNA seq data, revealed significantly higher GPCR levels in the GBM group (Figure 3F).

Subsequently, T cells were reclustered, leading to the identification of 11 T sub-clusters (Figure 3G). Cluster eight and Cluster 10 exhibited significantly higher GPCR scores (Figure 3H). Pseudo-time analysis demonstrated that T cells had three stages, and there was a transition of T cell clusters from low GPCR to high GPCR levels (Figure 3I).

**FIGURE 4:**
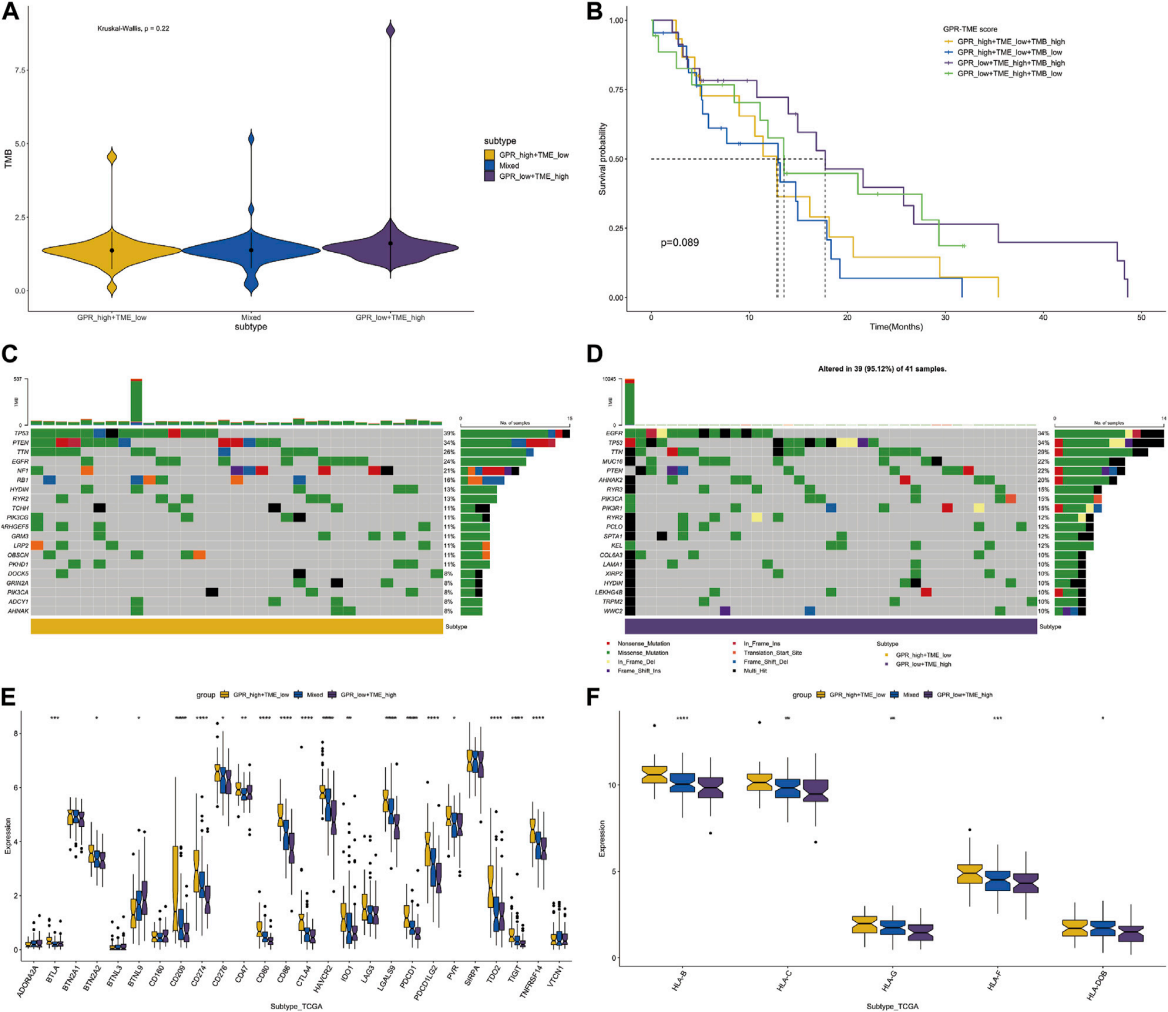
The classifier with GPCR and TMB in predicting survival probability in GBM **(A)** The TMB levels between the GPCR_low + TME_high and GPCR_high + TME_low groups **(B)** The survival probability among the four groups **(C–D)** The heatmap depicts the mutated genes between the GPCR_low + TME_high and GPCR_high + TME_low groups. The percentage of gene mutation is listed **(E)** The expression levels of immune check points are compared among the groups **(F)** The expression levels of HLA molecules are compared among the groups.

Considering the differences in GPCR scores between tumor and immune cells, we further compared the tumor mutation burden (TMB) level between different groups. Although not statistically significant, the TMB level was higher in the GPCR_low + TME_high group compared to the GPCR_high + TME_low group (Figure 4A). Consequently, the classifier based on GPCR level and TME level was unable to differentiate the survival status among these groups, although a trend was observed between the GPCR_low + TME_high group and the GPCR_high + TME_low group (Figure 4B). We then analyzed gene mutations between the two groups. In the GPCR_high + TME_low group (Figure 4C), the mutation level was lower compared to the GPCR_low + TME_high group (Figure 4D). The mutation percentage of each gene is also listed in Figures 4C,D. In the GPCR_low + TME_high group, the top five mutated genes were TP53 (39%), PTEN (34%), TTN (26%), EGFR (24%), and NF1 (21%), while in the GPCR_high + TME_low group, the top five mutated genes were EGFR (34%), TP53 (34%), TTN (29%), MUC16 (22%), and PTEN (22%). Moreover, expression of most immune checkpoints and HLA molecules was significantly higher in the GPCR_high + TME_low group, including BTLA, CD209, CD274, CD80, CD86, CTLA4, and PDCD1. Regarding the expression of HLA molecules, their levels were significantly higher in the GPCR_high + TME_low group.

## 4 Discussion

Previous studies have highlighted the critical roles of the TME in cancer (Sharma et al., 2019; Deng et al., 2020; Miyazaki et al., 2020; Qu et al., 2021), particularly in the heterogeneity and biological features of different TMEs. However, the precise mechanism by which GPCRs affect the TME remains a significant area of investigation. In this study, we investigated the expression profile of GPCRs in glioblastoma using both tumor bulk and single-cell RNA sequencing to evaluate their abundance and their association with TME, aiming to determine if they were a prognostic classifier for GBM.

Previous studies have also highlighted GPCRs as influential components of immune cells in various cancers (Zhang et al., 2010). GPCRs exert their function through ligand binding and direct cell-cell interactions (Yang et al., 2020). Notably, a series of adhesion GPCRs exhibit high expression levels in GBM tissues (Stephan et al., 2021), which aligns with our study (Figure 1A). GPCR160 is upregulated in U251 and H4 glioma cell lines, and its downregulation with siRNA has led to inhibited glioma cell proliferation, reduced migration, induced apoptosis, and decreased EMT biomarkers (Abbas et al., 2022). Similarly, miRNA-449a-mediated inhibition of GPCR158 suppressed glioma proliferation, particularly in high-grade glioma, while knockdown of GPCR158 increased the proliferation, migration, and sphere formation in GBM cell lines (Li et al., 2018).

Although there are currently no approved therapies targeting GPCRs in GBM, their unique characteristics, including high expression levels in GBM tissues and their ability to pass through the blood-brain barrier, make them promising targets for pharmaceutical intervention. Through multiple cox analysis, we identified CRCP as an independent risk factor in GBM and a potential novel target for treating glioblastoma.

Employing GSEA, we observed that high GPCR levels were associated with coagulation and complement system pathways, while low GPCR levels were negatively correlated with oxidative phosphorylation. Moreover, the TME high group showed a negative correlation with the complement and coagulation cascades, while the TME low group displayed a negative association with the p53 signaling pathway (Supp Fig 2). Abnormal coagulation is a pathology recently found in different cancers (Maruyama et al., 2022), which is related to the tumor thrombosis (Dirix et al., 2022; Sun et al., 2022). Our study may propose a new function of GPCR in the pathology of abnormal coagulation, which deserves further experiments.

Examining T cells based on their GPCR levels, we found that only those with high GPCR levels exhibited significant cell-cell interactions with tissue stem cells via PTN/NCL, while this connection was absent in T cells with low GPCR levels. Additionally, T cells with high GPCR levels demonstrated a connection with macrophages through Tnf/Tnfrsf1b, which was not observed in T cells with low GPCR levels. Studies in mice have suggested that Tnfrsf1b plays a role in protecting neurons from apoptosis by stimulating antioxidative pathways and inhibiting inflammatory pathways (Iosif et al., 2008). TNFα, a pro-inflammatory cytokine highly expressed in GBM, can bind to two receptors: TNFRSF1A and TNFRSF1B. Depletion of Tnfrsf1a can lead to neuroblast and subventricular zone cell proliferation (Iosif et al., 2008). Using cellchat analysis, we found that macrophages secreted TNF to T cells, which then bound to TNF receptors. This is the first time that GPCR has been linked to TNF in glioma. As CRCP encodes the protein which is a component of calcitonin gene-related peptide (CGRP) receptor and CGRP has been reported in neuroendocrine tumors, however, CRCP itself has not been mentioned by previous studies (Song et al., 2012; Kuo et al., 2022). Additionally, TNFα is thought to be a triggering factor in thrombus formation via activation of the complement system (Page et al., 2018). It might be proposed that increased TNFα in macrophage contributes to the thrombus in glioma (Czap et al., 2019; Kaptein et al., 2022). However, the intricate relationship between GPCR, TNF, and coagulation in glioma requires further investigation in future studies.

We also employed Proteomaps to examine protein functions within each cluster and observed that GPCR genes were prominently enriched among the up-regulated genes in the GPCR low group, regardless of immune therapy response. However, these GPCR genes were absent among the down-regulated genes in both the GPCR high and no response groups. This implies that targeting GPCR could serve as a potential strategy for GBM (Supp Fig 3).

Recent studies have associated GPCRs, particularly the prostanoid receptor family, including EP receptor 1, EP2, EP3, and EP4, with cancer immune evasion, where PGE2, the most prevalent prostaglandin in cancers (Wu et al., 2019). PGE2 secreted from cancer cells is associated with increased FOXP3 expression in Treg cells (Baratelli et al., 2005), a key component of the immune-suppressive environment. Apart from Tregs, PGE2 has been observed to recruit additional MDSCs (Obermajer and Kalinski, 2012) and deactivate CD8 T cells (Song et al., 2012). Hence, these GPCRs (EP receptors) present ideal targets for tumor immune therapy.

The study has some limitations that warrant attention. Firstly, the bulk-seq data (TCGA-GBM and CGGA) were obtained from public databases with a limited sample size. Although our in-house scRNA-seq data originated from surgical samples, only two brain tissues were included. Further research with larger sample sizes from multiple centers is crucial to validate our current results. Secondly, for comparison with the glioblastoma sample, we used meningioma as the control. Since it was unfeasible to acquire completely normal brain tissue for this study, meningioma, mostly a benign tumor with fewer invasive and invaded cells, was utilized. Lastly, the functions and potential molecular mechanisms of GPCR genes are highly intricate, necessitating additional experimental verification and the development of pharmaceutical drugs using cell and animal models.

## 5 Conclusion

To conclude, our study combines the analysis of publicly available bulk RNA-seq data with in-house scRNA-seq data to uncover GPCR-related genes and their contributions to the immune and survival phenotypes of GBM. These findings have the potential to yield valuable biomarkers and therapeutic targets for GBM.

## Data Availability

The data presented in the study are deposited in the OMIX repository, accession number OMIX004539.
