# Supplementary material for: A novel classifier combining G protein-coupled receptors and the tumor microenvironment is associated with survival status in glioblastoma
Source: Front Pharmacol. 2023 Jul 25;14:1093263. doi: 10.3389/fphar.2023.1093263 (PMC10407249; doi:10.3389/fphar.2023.1093263)
Supplement: Supplementary file 1 [file DataSheet1.docx]

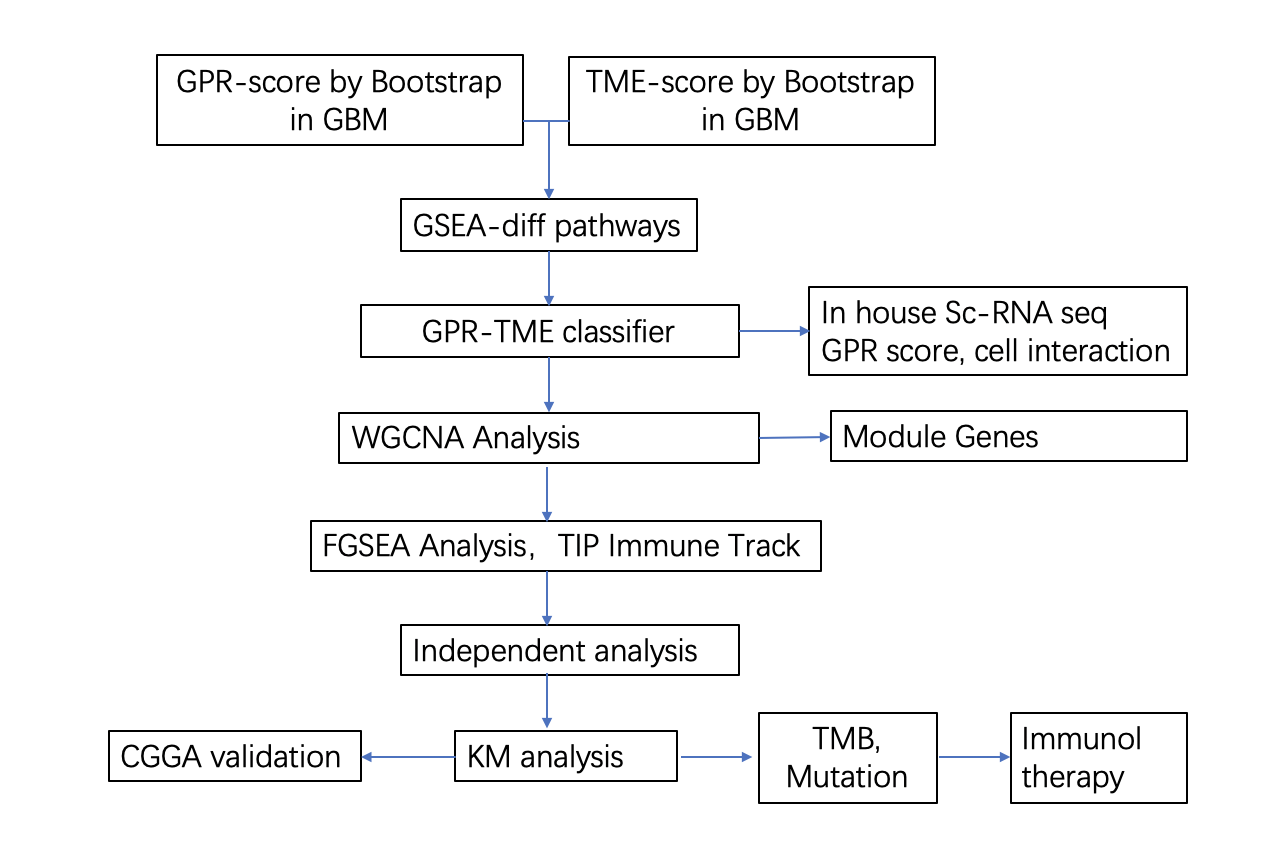


Supp Figure 1.


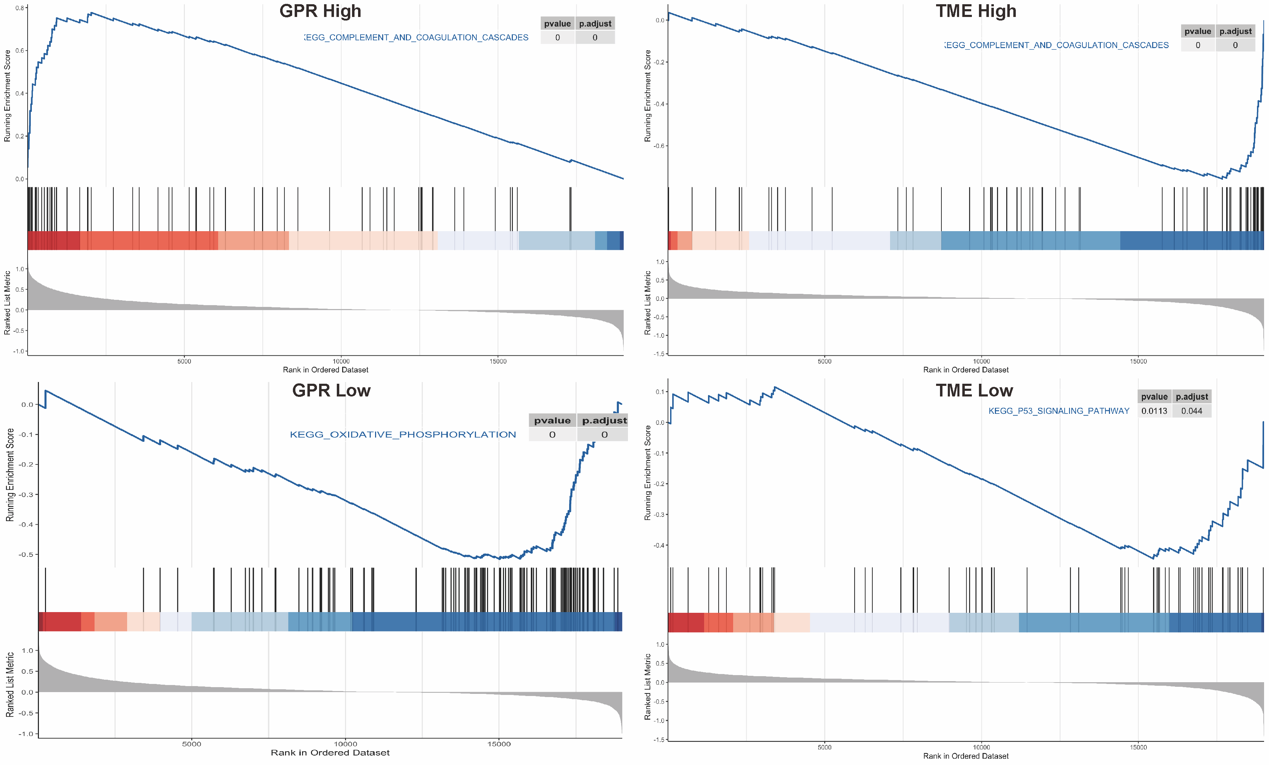


Supp Figure 2.


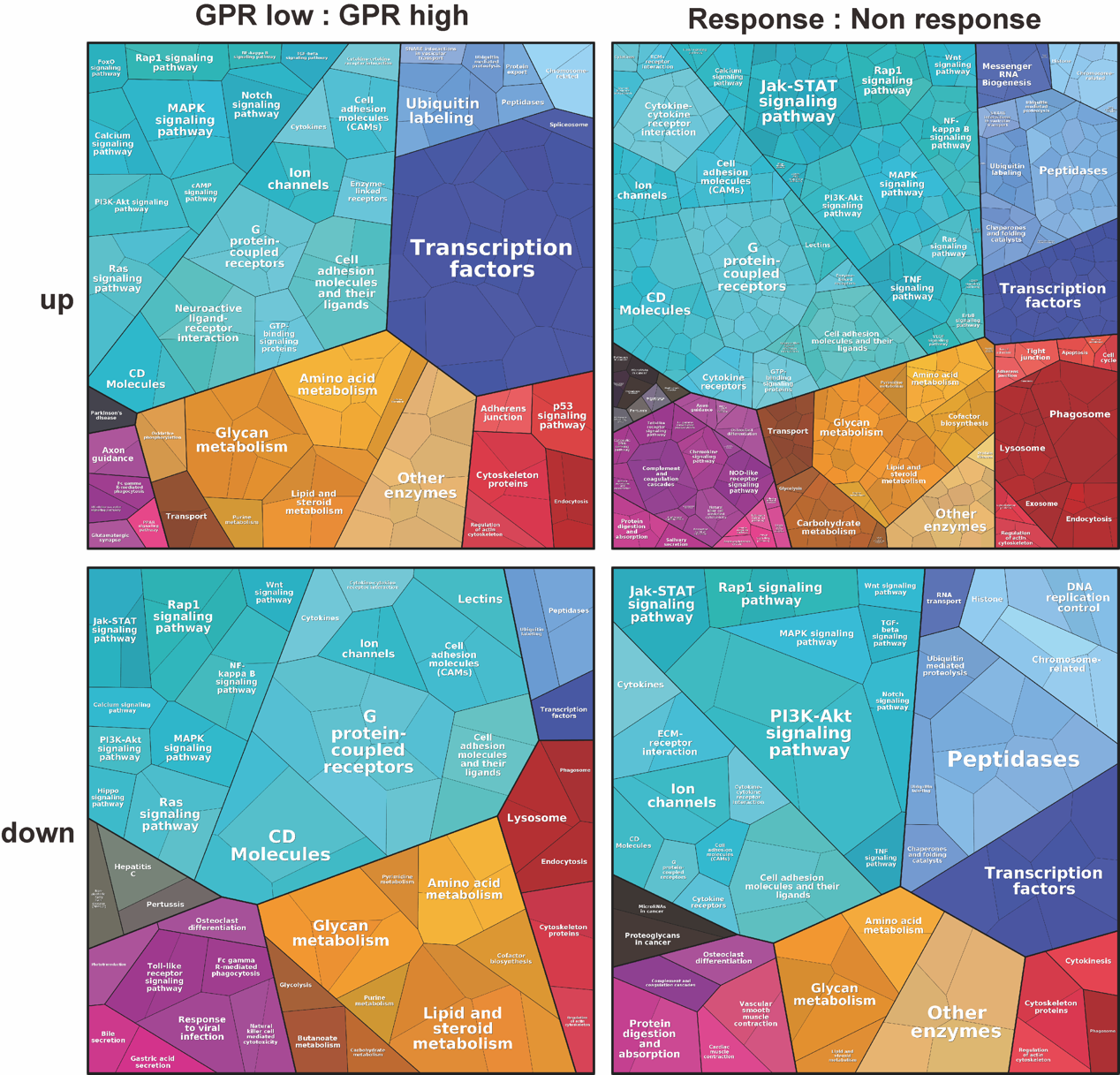


Supp Figure 3.

The attached file can be obtained at

https://www.jianguoyun.com/c/sd/166473d/30a6be2ee7e3b18f#from=https%3A%2F%2Fwww.jianguoyun.com%2Fc%2Fsd%2F166473d%2F30a6be2ee7e3b18f
